# Physiology, functional genomics, and proteomics of *Verruconatronum alginivorum* gen. nov.*,* sp. nov.*,* the first isolated haloalkaliphile within *Verrucomicrobiota,* representing a new family, *Verruconatronumaceae* fam. nov.

**DOI:** 10.1128/aem.00475-26

**Published:** 2026-05-11

**Authors:** Dimitry Y. Sorokin, Varada Khot, Alexander Y. Merkel, Damon Mosier, Nicole J. Bale, Michel Koenen, Marc Strous

**Affiliations:** 1 Winogradsky Institute of Microbiology, Research Centre of Biotechnology, Russian Academy of Sciences 54744, Moscow, Russia; 2 Department of Earth, Energy, and Environment, Faculty of Science, University of Calgary 685736, Calgary, Canada; 3 Institute of Biodiversity, Faculty of Biological Sciences, Cluster of Excellence Balance of the Microverse, Friedrich Schiller University Jena 9378, Jena, Germany; 4 Department of Marine Microbiology and Biogeochemistry, NIOZ Royal Netherlands Institute for Sea Research, Den Burg, Texel, the Netherlands; Kyoto University, Kyoto, Japan

**Keywords:** alginate, soda lakes, haloalkaliphilic, polysaccharide lyases, *Opitutales*, *Verrucomicrobiota*

## Abstract

Despite the successful cultivation of many microbes from rich bacterial communities inhabiting alkaline soda lakes, members of the bacterial phylum *Verrucomicrobiota* have so far been detected only through metagenomics. Here, we used alginate as a selective substrate to enrich and isolate two strains of haloalkaliphilic *Verrucomicrobiota*. The isolates share identical 16S rRNA gene sequences representing a new genus lineage, and, together with other metagenome assembled genomes, a new family within *Opitutales*. Cells of strains AB-alg1^T^ (from soda lakes) and AB-alg4 (from soda solonchak soils) are small and motile cocci forming submerged colonies in soft alginate agar. They are saccharolytic heterotrophs growing aerobically on polysaccharides (alginate, starch, and inulin) and sugars (glucose, fructose, mannose, sucrose, melezitose, maltose, and cellobiose). They also grow anaerobically by fermentation of alginate and D-mannose and by coupling incomplete denitrification to oxidation of alginate. Both isolates are obligately alkaliphilic and moderately salt-tolerant. The dominant membrane phospholipids include phosphatidylcholines and diphosphatidylglycerols (cardiolipins). The genome of AB-alg1^T^ features polysaccharide lyases of the PL6, 7, 15, 17, 38, and 39 families for depolymerization of alginate. Based on distinct phenotype and phylogeny, we propose classification of strains AB-alg1^T^ (JCM 35393^T^=UQM 41574^T^) and AB-alg4 as *Verruconatronum alginivorum* gen. nov., sp. nov. within a new family *Verruconatronumaceae*.

**IMPORTANCE:**

Alkaline soda lakes and soils are extreme habitats dominated by obligate haloalkaliphic prokaryotes, some of which can produce alkali- and salt-stable polysaccharide-degrading exoenzymes useful for industrial and domestic applications. However, so far, little was known about the microbial potential for mineralization of acidic polysaccharides, such as alginate, in these habitats. The described isolates are the first representatives of a new family within the phylum *Verrucomicrobiota* specializing in the degradation of alginate and related polysaccharides. We present the key enzymatic machinery for alginate breakdown. These enzymes are high-pH tolerant and have potential for industry applications, for example, in washing powders and biomass waste recycling. Furthermore, the new family is one of the most abundant taxa in alkaline environments, and these environments are not known to harbor signature alginate producing biota, such as brown algae. This way, our study opens a new window on polysaccharide turnover in alkaline environments.

## INTRODUCTION

Soda lakes and soda solonchak soils are unique alkaline and saline habitats characterized by stable high pH due to the presence of molar concentrations of sodium carbonate and bicarbonate, selective for obligate haloalkaliphilic prokaryotes. The prokaryotic communities of soda lakes—and, to a lesser extent, those of soda solonchak soils—have been intensively studied during the last several decades in Central Asia (southern Siberia, north-eastern Mongolia, and Inner Mongolia), East African Rift Valley, California and Nevada (US), and British Columbia in Canada. Culturing and molecular ecology investigations revealed taxonomically diverse microbial communities active in carbon, sulfur, and nitrogen cycling present in these soda lakes (1–9).

Our recent efforts focused on the degradation of polysaccharides in soda lakes resulted in the isolation of a range of extremely halophilic natronoarchaea capable of growth with chitin, cellulose, and many other polysaccharides of neutral sugars (3, 10–12). However, these organisms were unable to degrade acidic polysaccharides based on uronic acids. Therefore, attempts continued to target acidic polysaccharides-utilizing bacteria in soda lakes and soda solonchak soils. Sodium hyaluronate (a linear heteropolymer of *N*-acetylglucosamine and glucuronic acid) turned out to be an extremely selective substrate for enrichment and isolation, at moderate salinity, of haloalkaliphilic bacteria belonging to the phylum *Planctomycetota,* described recently as a new genus *Natronomicrosphaera* within the family *Phycisphaeraceae* (13).

Here, we focused on the acidic polysaccharide alginate, a heteropolymer of mannuronic and guluronic acids. Growth on alginate requires a specialized pathway comprised of extracellular polysaccharide lyases, glycoside hydrolases, and final reductive cleavage via the Kdu-Kdg system, yielding pyruvate and 3-phosphoglycerate (14–18). Alginate turnover is a hallmark of marine ecosystems where brown algae are important producers. Currently, not much is known about alginate turnover in alkaline soda lakes. Even though brown algae do not grow there, there might be other sources of alginate. Alkaline soda lakes are open systems, are visited by migrating birds, receive terrigenic matter via runoff, and experience occasional blooms of other eukaryotic algae. In any case, high pH and alkalinity enrichment cultures with alginate as the only carbon and energy source led in the enrichment and isolation of members of the phylum *Verrucomicrobiota* which, so far, lacked any haloalkaliphilic representative in pure culture. The isolates formed a new genus and family within the order *Opitutales*, representing one of the most abundant taxa found in alkaline soda lakes. This paper describes their phylogeny, physiology, membrane lipid composition, and results of functional genome analysis.

## MATERIALS AND METHODS

### Inoculum and enrichment conditions

The top 1–2 cm layer of oxic sediments and bottom brines (1:3, vol/vol) were collected from six moderately saline soda lakes located in the south of Kulunda Steppe (Altai region, Russia; N52^o^06′/ E79^o^09′; N51^o^37′/ E79^o^50′; N51^o^39′/ E79^o^48′; N51^o^40′/ E79^o^54′- E79^o^54′) in July 2022. The salt concentration of the brines ranged from 50 to 150 g L^−1^, the pH from 10.1 to 10.6, and the carbonate alkalinity from 0.5 to 1.8 M. Salinity and pH of the brines were measured using a WTW field potentiometer-conductometer (Munich, Germany). The total salinity values were additionally verified gravimetrically in the laboratory after the brine filtration through 0.45 μm filters, and the average of the values obtained by the two methods was used. Soluble carbonate alkalinity was determined in the field by a two-step titration with 1 M HCl: down to pH 8.0 (carbonate alkalinity) followed by further titration to pH 4.0 (the bicarbonate formed from carbonate + native bicarbonate alkalinity). The same technique was applied for soda solonchak soils, except that the samples were first resuspended (1:5, vol/vol) in distilled water, to dissolve the soluble salts, and the resulting brine was filtered first through a cotton lug followed by 0.45 μm membrane filtration.

Five milliliters of the sediment: brine suspensions from individual samples were mixed in equal proportions in a 50 mL Falcon tube, homogenized by vigorous shaking, and incubated statically for 1 h to allow sedimentation of coarse heavy particles. The residual top 10 mL fraction containing fine particle suspension was then used as inoculum (5%, vol/vol). The inoculum from three soda solonchak samples (collected in the same area as the lakes) was prepared by the same way: 1 g (dry weight) of each soil was mixed and homogenized in a sterile mortar to a fine powder and suspended in sterile 0.5 M NaHCO_3_ 1:5 (wt/vol). After vigorous shaking, heavy sand material was sedimented, and the residual fine fraction was used as inoculum (5%, vol/vol).

The enrichment medium was based on sodium carbonate/bicarbonate buffer containing 0.1 M NaHCO_3_, 0.2 M Na_2_CO_3_, 0.1 M NaСl, 1 g L^−1^ K_2_HPO_4_, and 20 mg L^−1^ yeast extract (final pH of 9.5 after autoclaving at 120°C). After sterilization, the base medium was supplemented with 1 mM magnesium sulfate, 4 mM NH_4_Cl, and 1 mL each of trace metal and vitamin mix (19, 20). 1 g L^−1^ sodium alginate from brown algae (Sigma-Aldrich, St Louis) was added from a 5% (wt/vol) stock solution as the carbon and energy source, and cultures were incubated at 30°C on a rotary shaker at 120 rpm in screw cap bottles until visible microscopic evidence of bacterial growth (after settling of sediment particles by low-speed centrifugation).

### Growth physiology

Optical density was measured with a Ultrospec 10 Cell density Meter (Cambridge, UK). pH was measured with a Jenway Codactivity & pH Meter 4330 (Essex, UK). Substrate profiling was performed in aerobic cultures at optimal pH/salt conditions and 30°C (see below, incubator from Edmund Bühler GmbH, Bodelshausen, Germany) in serum bottles (5 mL/23 mL) with rubber stoppers to prevent medium evaporation, shaken at 150 rpm. Substrates were added from 10% sterile stocks (polysaccharides—autoclaved, sugars—filter-sterilized). The following sugars were obtained from Sigma-Aldrich (St. Louis, USA): Sodium heparin sulfate from porcine mucosa, agarose, gellan, pectins from cirrus and apple, polygalacturonate, dextran from *Leuconostoc*, birch and beech xylans, and shrimp chitin. Xyloglucan from tamarind, arabinan from sugar beet, potato galactan, inulin from chicory, β-mannan from ivory nut were from Megazyme (Warwick, Ireland). Sodium hyaluronate (1–2 MDa) from synovial liquid, chondroitin sulfate A from bovina cartilage, and fucoidan from Fucus algae were from Biosynth (Bratislava, Slovakia). Growth intensity was estimated by the increase of OD_600_ in comparison to the cell-free controls, either directly in case of soluble substrates or after precipitation of insoluble polysaccharides either by gravity or by a low speed centrifugation. Salinity tolerance was examined in a sodium carbonate/bicarbonate buffer (pH of 9.5) containing 0.1–2.5 M Na^+^. The growth pH range was studied using 50 mM HEPES/50 mM potassium phosphate/0.6 M NaCl for the neutral range from 6 to 8, 0.5 M bicarbonate/1 M NaCl for the intermediate pH 8–8.5, and bicarbonate/carbonate with 0.6 M total Na^+^ for the alkaline range from pH 8.5–10.5. The actual pH was measured at the start and at the end of the experiments since it was drifting substantially from the starting set up values at the lowest and highest extremes. These incubations were performed at 30°C on a rotary shaker at 150 rpm. All other growth tests (temperature range, substrate utilization profiling, anaerobic growth) were done in carbonate medium with 0.6 M total Na^+^ and pH 9.5. Anoxic medium was prepared using a sterile Argon gas flushing-cold boiling evacuation system (3 cycles) in 23 mL serum bottles with 10 mL medium closed with butyl rubber stoppers. The medium was made anaerobic by a final addition of 0.1 mM of filter-sterilized Na_2_S. Biomass growth was monitored by increase in OD_600_. A rotary shaker (150 rpm) was used for aerobic incubations, while anaerobic growth was conducted in static incubations. The nitrous oxide concentration in the gas phase of anaerobic cultures was measured after complete nitrite consumption using a Varian 2000 GC equipped with a Porapaq-Q column (80/100 mesh) and Ni-63 electron capture detector (ECD).

### Microscopy and chemotaxonomy

Phase contrast microscopy (Zeiss Axioplan Imaging 2 microscope, Göttingen, Germany) was applied for routine checks. Electron microscopy was used to examine flagellation and cell ultrastructural organization. For the latter, the cells were centrifuged, resuspended in 0.5 M NaCl, fixed with *p*-formaldehyde (final concentration 3%, vol/vol) at room temperature for 2 h, and then washed again with the same NaCl solution. For whole cell imaging, the fixed cells were positively contrasted with 1% (wt/vol) uranyl acetate. For thin sectioning, additional fixation was done by 1% (wt/vol) OsO_4_. After washing in 0.5 M NaCl (pH 7), the cells were embedded in 2% agarose, dehydrated in acetone, and embedded in Araldite. Thin sections were sequentially stained with 2% uranyl acetate and 2% lead citrate for 20 min each and examined with a Jeol JEM-1400 electron microscope (Japan).

Membrane polar lipids and respiratory quinones were extracted with a modified Bligh-Dyer procedure from freeze-dried cells grown at 30°C at 0.6 M total Na^+^, pH 9.5 with Hyl until the late exponential growth phase and analyzed by Ultra High Pressure Liquid Chromatography-High Resolution Mass Spectrometry (UHPLC-HRMS^n^), as described previously (21). For the core fatty acids profiling, the cells were hydrolyzed in HCl/MeOH (1.5 N) and extracted with dichloromethane and further processed, analyzed, and lipids identified as described (21, 22).

### Genome sequencing and phylogenomic analysis

The strains were first identified by the Sanger sequencing of their nearly complete (1,452 nt) 16S rRNA genes amplified with 3 forward and one reversed primer sets (Bacterial 8f, 343f, 1114f, and 517r). For short read DNA sequencing of strain AB-alg1^T^, genomic DNA was extracted with the FastDNA SPIN Kit for Soil (MP Biomedicals, Santa Ana, CA, United States). Shotgun library preparation was performed using the Illumina DNA Prep (M) Tagmentation kit, and sequencing was performed on a NovaSeq 6000 (2 × 151 bp) system (Illumina, San Diego, CA, USA). For the long reads, high molecular weight genomic DNA was extracted and purified by the phenol-chloroform method in the presence of CTAB according to the JGI protocol v.3. The further preparation of genomic libraries and Nanopore sequencing was performed as described previously (23). The contigs were assembled with Unicycler v.0.5.0 (24) and submitted for automatic annotation to the PGAP (25) in GenBank. Genome completeness and contamination were assessed using CheckM v1.2.4 (26). For placement of the AB-alg1 genome into a species tree, 120 single copy conserved bacterial markers were identified and aligned according to the Genome Taxonomy Database release 220 (27) from 703 genomes, including an outgroup of *Planctomycetota* aligned using GTDB-Tk v2.4.1 (28) “identify” and “align” commands. These genomes were manually selected from the GTDB RS226 database to maximize the quality (based on completeness and contamination) of representative genomes for all class-level groups from the phylum *Verrucomicrobiota*, all order-level groups from the class *Verrucomicrobiia* and all family-level groups from the order *Opitutales*. A species tree was built using the resulting alignment with IQ-TREE2 v2.2.0.3 (29) with fast model selection via ModelFinder (20) and ultrafast bootstrap approximation with 1,000 bootstraps (30). Relative evolutionary divergence (RED) value was calculated using GTDB-Tk v2.4.1 “de_novo” workflow (--gamma --prot_model LG --outgroup_taxon p__Spirochaetota) and PhyloRank (https://github.com/dparks1134/PhyloRank). Percentage of conserved proteins (POCP) was calculated using POCP-nf v2.3.6 (31). A search among high-throughput sequencing data for 16S rRNA gene regions was performed by IMNGS (32) using 99% similarity threshold and 200 bp as a minimum size threshold. The presence of MAGs in public shotgun metagenome data sets was analyzed by Sandpiper 1.0.1 (33) using f__T3Sed10-336 as query for search by GTDB RS226 taxonomy.

### Ancestral reconstruction analyses

Genes were predicted and annotated from the same genomes used for the phylogenetic placement of AB-alg1 using MetaERG v2.5 (34). Seventy-four representative genomes were subsampled from the species tree. This included all the genomes, with a completeness ≥90% and contamination ≤5%, from the *T3Sed10-336* family, 3 representatives from every other family in the *Opitutales* order, 2 representatives from every other order from the *Verrucomicrobiae* class, 1 representative from every other class in *Verrucomicrobiota* phylum, and 5 representatives from the *Planctomycetota* phylum as an outgroup (Table S1). A multiple sequence alignment was created for these genomes based on 120 single copy conserved bacterial markers according to the Genome Taxonomy Database (27) from 74 genomes using GTDB-Tk v2.4.0 (28) “identify” and “align” commands. A species tree was built using the resulting alignment with IQ-TREE2 v2.2.0.3 (29) with fast model selection via ModelFinder (35) and ultrafast bootstrap approximation with 1,000 bootstraps (30) as well as approximate likelihood-ratio test for branches (36). Orthogroups were formed using genes from these 74 genomes using Orthofinder v3.1 (37) and aligned within Orthofinder using famsa v2.4.1 (38). Gene trees were then constructed for orthogroups containing genes from AB-alg1 identified to participate in the alginate metabolism using IQ-TREE2 v2.2.0.3 (29). First, a guide tree was created using the model “LG+F+G,” followed by a posterior mean site frequency (PMSF) model with “LG+C20+G” and 5,000 ultrafast bootstraps (39). Gene trees were reconciled with the species tree using AleRax v1.4 (40) with either 100 or 500 gene tree samples. For further analyses, only genes with a ≥70% probability were considered present at ancestral nodes and transfer events had to be seen in at least 50 bootstraps. Reconciled gene trees were visualized using ThirdKind (41) and the ggtree package in R (42).

### Functional genomic and proteomics analyses

Polysaccharide lyase families which include characterized endo/exo-alginate hydrolyzing activities documented in the CAZy database (43, 44) were identified using the dbCAN3 portal scan of the translated proteome (45). The identified proteins were manually checked for the presence of signal peptides using the Signal 6.0 server (46). Transmembrane sequences were predicted using TMHMM 2.0 (47). Similar and experimentally validated proteins were identified using Blast against the UniProt database (48). Domain architecture was inspected with the HMMER 3.4 portal (49) and InterPro 106.0 (50). Inference of the metabolic pathway for uronate hydrolysis was done by UniProt Blast search (51) using model proteins from an alginate-metabolizing *Flavobacterium* sp (16) and *Vibrio pelagicus* (15). Nitric oxide reductase was identified using custom HMM models (52).

Proteomics was performed for strain AB-alg1^T^ grown on alginate, mannose, and sucrose. Cells were harvested for proteomics after three consecutive transfers on each of these substrates at mid-exponential growth phase. The detailed workflow protocol was as described previously (23). Briefly, the freeze-dried cells were lyzed in an SDS/DTT buffer at 95°C, and the total proteins were separated on a Microcon spin filter and hydrolyzed by trypsin. Peptides were desalted with C18 ZipTips (Millipore Sigma, Burlington, USA) according to manufacturer’s protocol and submitted to LC-MS/MS. Data were collected using an Easy-nLC 1200 system coupled to a QExactive Plus (ThermoFisher Scientific, Waltham, USA). Approximately 1.5 µg of each sample was injected onto a 300 μm × 5 mm PepMap Neo Trap Cartridge (C18, 5 μm particle size, 100 Å pore size, ThermoFisher Scientific, Waltham, USA) and then separated by reverse phase chromatography using an EASY-Spray HPLC analytical column (75 μm × 75 cm, C18, 2 μm particle size, 100 Å pore size, ThermoFisher Scientific, Waltham, USA) at a flow rate of 225 nL/min at 40°C. Mobile Phase A consisted of 0.1% (vol/vol) formic acid in water, while Mobile Phase B consisted of 0.1% (vol/vol) formic acid in 80:20 acetonitrile:water (vol/vol). Separation was carried out using a multistep 140 min gradient from 2%–31% B for 102 min; 31%–50% B for 18 min, followed by a wash step at fixed 99% B for 20 min. Mass spec data were collected in positive mode at 1,800 V using a data-dependent acquisition (DDA) routine. Full MS scans were performed in the Orbitrap at 70,000 resolution (at *m*/*z* 200) for the 380–1,600 *m*/*z* range, with a maximum injection time of 200 ms and AGC target set at 3e6. The Top 15 precursors charged 2–8+ were selectively isolated (quadrupole isolation window of 1.2 Th) for fragmentation through HCD at a normalized collision energy of 25. MS2 scans were performed in the Orbitrap at 17,500 resolution (at *m*/*z* 200) at the 200–2,000 *m*/*z* range, with maximum injection time of 100 ms and AGC target set at 5e4. The dynamic exclusion window was set to ignore ions within 20 s and 10 ppm tolerance. Spectrum Peptide Matching was performed using FragPipe (53–56), with the predicted protein sequences of strain AB-alg1^T^ as the database. The protein relative abundances under each condition, reported in Table S3, were calculated based on spectral counts and corrected for amino acid length. Because we had only one replicate per condition, we estimated the standard deviation of the protein relative abundances by assuming that for most proteins in our data set, expression was unaffected by treatment. Thus, we collected all proteins that had non-zero relative abundances with both alginate and sucrose (*n* = 1,818) and plotted the difference of the log-transformed protein abundances in the two treatments vs the log transformed means of the treatments in a Bland-Altman plot. Note that we did not use the maltose data for this analysis because we detected a much smaller number of proteins in that analysis (see Table S3). The Bland-Altman plot showed that the standard deviation was independent of the mean (after log transforming the data, see Fig. 4, inset), so it could be applied to all proteins in our data set. The standard deviation of the log transformed relative protein abundance was then estimated to be 0.5703 based on the standard deviation of the differences in expression between treatment and control. The standard deviation was used to estimate the significance of differences in relative protein abundance between treatment and control using two-sided *P*-values based on *z*-scores, corrected for large *n* using Benjamini-Hochberg correction. Only 17 out of 1,818 genes showed a significant difference between treatment and control, showing that our assumption that for most proteins, expression was unaffected by treatment, was correct. These calculations were performed in Python, with help from Microsoft Copilot. Scripts are provided in Table S6.

## RESULTS AND DISCUSSION

### Isolation of pure cultures

Both soda lake and soda soil primary alginate enrichments showed growth (in turbidity and cell density increase) within 10 days and were dominated by small motile cocci. They were first passed two times at 1:100 dilution to ensure growth reproducibility and to obtain sediment-free cultures. Unfortunately, this led to the co-enrichment of flagellated protozoa, which were able to rapidly decrease the bacterial cell density. The protozoa were successfully eliminated by serial dilutions up to (10^−10^) in the same medium. However, both enrichments still contained a rod-shaped phenotype and surface plating resulted in colony formation only from the latter. Therefore, considering our previous experience with the hyaluronate-utilizing *Natronomicrococcus* from the same habitats, attempts were made to achieve colonial growth of the dominant micrococci by mixing the culture into soft agar. No colonies formed in 1% agar, while use of 0.6%–0.8% agar led to formation of separate colonies of the target cocci from both enrichments after inoculation with up to (10^−8^) diluted liquid cultures. Transfer of a colony into liquid medium with alginate resulted in vigorous growth of morphologically uniform motile micrococci, and the purity of two cultures was confirmed by the nearly complete 16S rRNA gene Sanger sequencing: the lake isolate was designated as AB-alg1^T^ and the soil culture—as AB-alg4. As their 16S rRNA gene sequences were identical, further work was mostly done with the type strain AB-alg1.

### Phylogenetic analysis, classification, and environmental distribution

Preliminary identification based on the 16S rRNA gene sequence analysis placed the isolates into a new lineage within the phylum *Verrucomicrobiota* which, so far, did not have any cultivated haloalkaliphilic representatives from soda habitats. The closest relatives among the cultivated members in this phylum were *Opitutus terrae* and other members of the order *Opitutales* (Table 1), with the percentage of 16S rRNA gene identity always below 90%.

**TABLE 1:** Comparative properties of strain AB-alg1^T^ with the type species of the related genera in the family *Opitutaceae^c^^,^^d^*

Property	AB-alg1^T^	Opitutus *terrae* (57)	*Rariglobus hedericola* (58)	*Cefaloticoccus* *primus* (59)	*Pelagicoccus* *mobilis* (60)	*Ereboglobus* *luteus* (61)	*Geminisphaera* *colitermitum* (62)	*Nibricoccus* *aquaticus* (63)
Colonies	White, hard^*a*^	White, granular^*a*^	White, circular	Cream	White	Yellow lenses	White	Yellow
Cells	Motile cocci	Motile cocci	Motile cocci	Nonmotile cocci	Motile cocci	Motile cocci	Motile diplococci	Nonmotile cocci
Relation to O_2_	Facultative anaerobe: fermentation NO_2_^−^>N_2_O	Obligate anaerobe: fermentation NO_3_^−^>NO_2_^−^	Obligate aerobe	Obligate aerobe	Obligate aerobe	Facultative anaerobe: fermentation	Obligate aerobe	Obligate aerobe
Growth substrates: polysaccharides	**Alginate**, starch, inulin	Starch, xylan, pectin	nd	nd	nd	Pectin	Starch	Dextrin
Growth substrates: sugars	Glucose, fructose, sucrose, mannose, melezitose, fucose, maltose, cellobiose	Glucose, fructose, sucrose, mannose, galactose, lactose, maltose, arabinose, cellobiose, melibiose, galacturonic acid	Glucose, fructose, glucuronamide	Glucose, fructose, mannose, sucrose, melibiose, maltose, cellobiose, lactose, raffinose, turanose, trehalose	nd (only acid production)	Glucose, fructose, galactose, lactose, maltose, arabinose, cellobiose, ribose, melibiose, trehalose, galacturonic acid	Glucose, galactose, maltose, cellobiose	Glucose. mannose, melibiose, rhamnose, maltose, cellobiose, galactose, gentibiose, lactose, lactulose, arabinose, raffinose, fucose, glucuronic acid, galacturonic acid
pH range (opt.)	8.2–10.3 (9.5)^*b*^	5.5–9 (7.5–8.0)	nr (neutrophile)	6.9–7.7 (7.0)	6.0–9.0 (7.5–8.0)	6.0–9.0 (7.0)	5.5–7.5 (7.0)	5.5–9.0 (7.0)
Salinity range (opt.), Na^+^ (M)	0.2–1.8 (0.4–0.6)	nd	0-0.1 (0)	0.1–0.25 (0.15)	nd	nd	0–0.25 (0)	0–0.1 (0)
N-source (apart from ammonium)	Urea, nitrate (growth, genomic)	Urea (genomic)	Urea, nitrate (genomic)	Urea (genomic)	Urea (urease test; genomic)	–	Urea (genomic)	Urea, nitrate (urease test, genomic)
Nitrogenase	–	–	–	–	–	–	+ (growth, genomic)	+ (activity, genomic)
Max. temperature (^o^C)	40	37	30	37	37	37	35	37
Catalase/oxidase tests	+/+	−/−	−/−	−/−	−/+	−/−	−/−	−/−
Intact membrane polar lipids	PC, DPG, PE, PG	nd	PE, PG, DPG	nd	nd	nd	nd	PE, PG, DPG
Predominant polar lipid fatty acids	*ai*C_15:0_, C_18:0_, C_16:0_	nd	C_14:0_, C_16:0_, *i*C_14:0_, *ai*C_15:0_ C_16:1*ω*5_	*ai*C_15:0_, *i*C_14:0_, C_16:0_, C_16:1*ω*5_	*ai*C_15:0_, C_16:0_, C_16:1*ω*7_	nd	*ai*C_15:0_, *i*C_15:0_	*i*C_14:0_, *ai*C_15:0_, C_16:0_, *i*C_16:0_
Respiratory lipoquinones	MK-7:7	nd	MK-7	nd	MK-7	nd	nd	MK-7
Genome size (Mbp)	6.0	6.0	4.1	2.4	7.5	4.2	5.2	4.7
G + C (%, whole genome)	59.3	65.5	60.6	63.0	53.5	59.7	60.5	62.0
Habitat	Soda lakes and soda solonchak soils	Paddy soil	Freshwater	Ants gut	Marine	Cockroach hindgut	Termite gut	Freshwater

^*a*^ Submerged in soft agar.

^*b*^ Actual measured values.

^*c*^ Lipids: PC, phosphatidylcholine; PE, phosphatidylethanolamine; PG, phosphatidylglycerol; DPG, diphosphatidylglycerol.

^*d*^ −, negative; nd, not detected.

**Fig 1:**
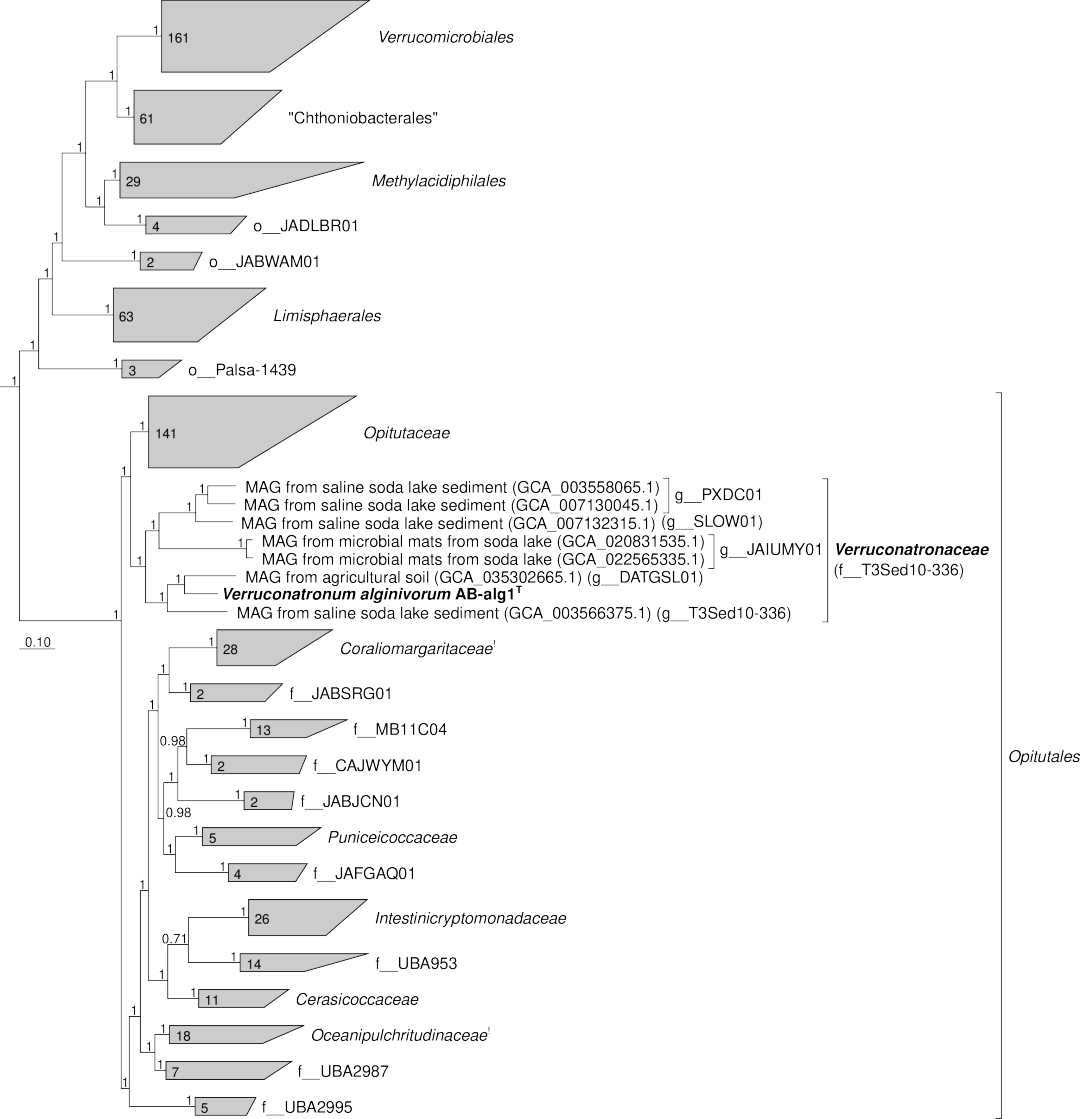
Phylogenetic placement of strain AB-alg1^T^ within the *Verrucomicrobiia* based on phylogenetic analysis of a concatenated 23,562 aa alignment of partial amino acid sequences of 120 bacterial conservative proteins by maximum likelihood inference. Bootstrap values are shown at the nodes (1 = 100%). Bar, 0.10 changes per position. Taxonomic designations are given according to the GTDB RS226. Clades with alkaliphilic representatives are indicated with an exclamation mark after the taxon name. All class-level groups from the phylum *Verrucomicrobiota* were included in theanalysis (not shown). Sequences of representatives of the phylum *Planctomycetota* were used for tree rooting.

DNA was extracted from the AB-alg1^T^ cells grown with alginate and after short read and long read sequencing, the genome was assembled into a single linear contig. The genome size was 6.01 Mb with a GC content of 59.4% and completeness and contamination of 99.32% and 1.69%, respectively (26). To clarify the phylogenetic position of the AB-alg1^T^ strain, we made a phylogenomic reconstruction based on 120 single copy marker genes (bac120) (27), but with a much larger alignment length (23,562 aa) and using maximum-likelihood instead of approximate maximum-likelihood. This reconstruction confirmed that this bacterium forms a deeply branching lineage within the *Opitutales,* which, together with several metagenome-assembled genomes (MAGs) from soda lakes, forms a separate family, sister to the type family *Opitutaceae* (Fig. 1). This phylogenetic lineage is designated in the GTDB release RS226 as T3Sed10-336 (the abbreviation originated from the “sediments of Tanatar-3 soda lake in Kulunda Steppe”) and has 100% bootstrap support in both our and the GTDB RS226 phylogenetic reconstructions. This lineage has the relative evolutionary divergence (RED) value 0.742, which is slightly below the median RED values for bacterial families in GTDB RS226 (0.762). AB-alg1^T^ represents a separate genus-level lineage in addition to which the T3Sed10-336 cluster also includes several genus-level lineages of MAGs from moderately saline soda lakes in the same area, g__PXDC01, g__SLOW01, and g__T3Sed10-336 (6) and from a similar type of soda lakes in British Columbia (Canada), g__JAIUMY01 (9). The AB-alg1^T^ strain and the MAG closest to it (GCA_035302665.1) have only 39.8% POCP. In addition, their joining node has a RED value of 0.869, which is significantly lower than the median RED values for bacterial genera in GTDB RS226 (0.92). Thus, the AB-alg1^T^ strain represents a standalone genus.

16S rRNA-based analysis using the IMNGS service showed that phylotypes related to AB-alg1 (>99% identity of the V4 region of the 16S rRNA gene) are found only as single or a few sequences in samples of two saline soda lakes: Mono Lake (USA, PRJNA387610) and Lake Gahai (China, PRJNA294836). At the same time, metagenome-based analysis using the Sandpiper resource (33) showed that phylotypes belonging to T3Sed10-336 family-level lineage are found in ~800 metagenomes. In natural habitats, T3Sed10-336 was most abundant (0.5%–3%) in alkaline lakes and soils, ranking it within the top 20 most abundant taxa in these environments. In one engineered environment, a photobioreactor inoculated with microbial mats from Canadian alkaline soda lakes (64), it made up 30% of the microbial community. Apparently, T3Sed10-336 fulfills a niche in these natural and engineered communities, which may include degradation of alginate and related acidic polysaccharides, such as hyaluronic acid and chondroitin.

### Cell morphology and chemotaxonomy

**Fig 2:**
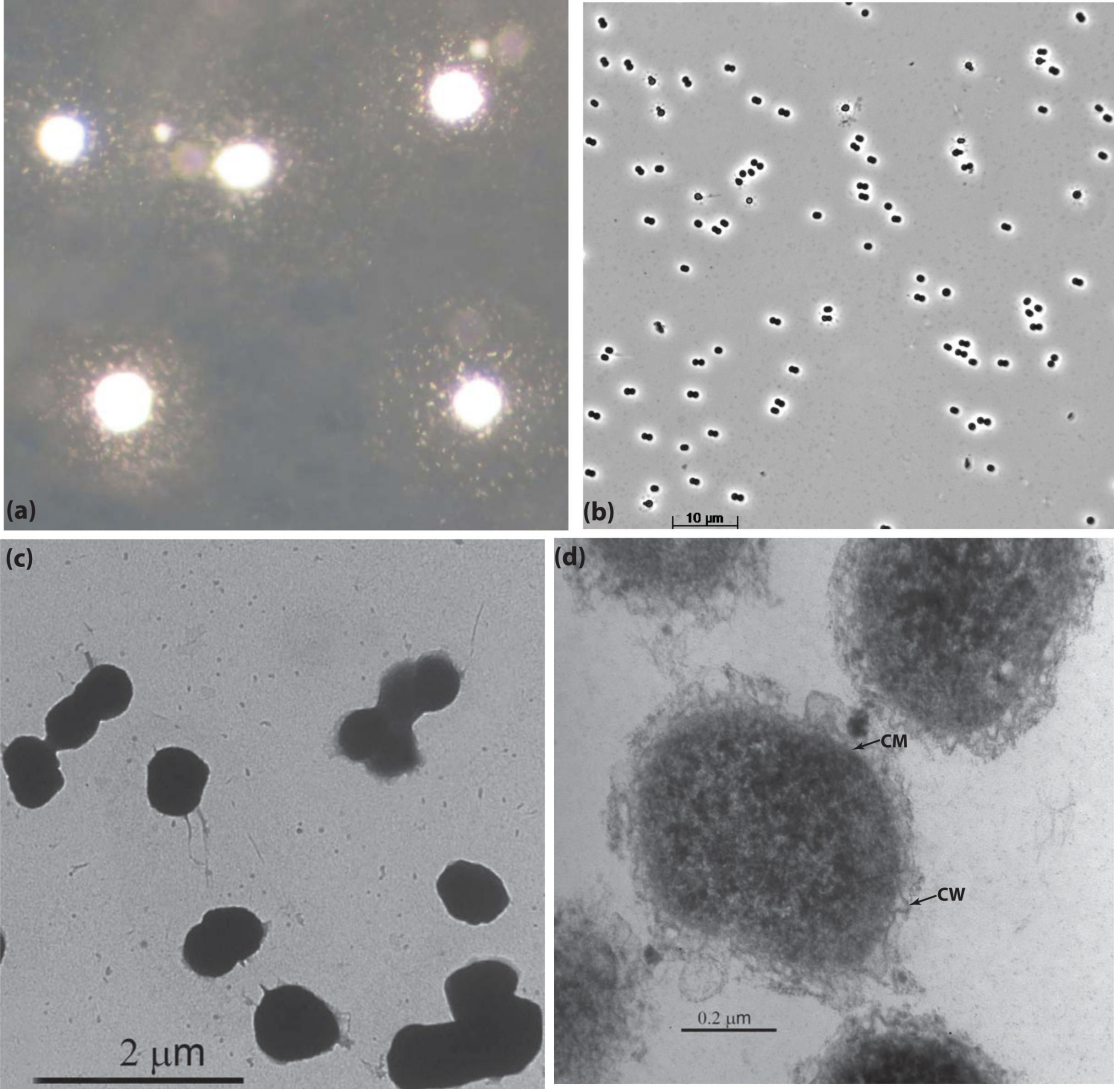
Colony (inside soft agar) (**a**) and cell morphology (**b–d**) of strain AB-alg1^T^ grown with alginate at 0.6 M total Na^+^, pH 9.5, and 30°C. (**b**) Phase contrast microphotograph, (**c**) transmission electron microscopy microphotograph, and (**d**) thin section electron microscopy microphotograph showing Gram-negative type of cell ultrastructure with an extended periplasm. CW, cell wall; CPM, cytoplasmic membrane.

Both isolates formed hard white colonies submerged in soft agar (Fig. 2a). In liquid cultures, the cells grew mostly in homogeneous suspension as small mono- or diplococci and were motile with 1–2 flagella (Fig. 2b and c). A major problem with the isolates was their rapid cell lysis in liquid cultures reaching stationary phase and culture storage at 4°C. The reason for this became obvious after electron microscopy of thin sections which showed a typical Gram-negative cell ultrastructure with a loose cell wall (Fig. 2d). Storage of exponentially growing cultures with 15% glycerol (vol/vol) at −70°C allowed for a long-term preservation of the isolates.

The major respiratory menaquinones detected were fully unsaturated MK-7:7 and its 2,3-epoxy-modification, which was identified based on the mass and similarity of its mass spectrum to published spectra (65). It is not clear, however, whether the epoxy-modification was an artifact of the extraction protocol or a natural component of the respiratory MK pool. The identified membrane phospholipids included phosphatidylcholines (PCs) and diphosphatidylglycerols (DPGs, also known as cardiolipins), with smaller amounts of phosphatidylethanolamines (PEs), lyso-PCs (which miss one fatty acid), phosphatidylglycerols (PGs), and acyl-PGs. The dominant polar lipid fatty acids (in order of abundance) were *anteiso*—C_15:0_, C_18:1ω9_, C_18:0_, and C_16:0_. Among those, the *anteiso-*C_15:0_ and C_16:0_ are also among the dominant fatty acids in other *Opitutaceae* genera, while the C_18_ fatty acids are absent in *Opitutaceae* but occur in other families of the *Opitutales* order (Table 1; Table S2).

### Growth physiology

Substrate profiling of strain AB-alg1^T^ and AB-alg4 demonstrated that the bacteria have an organoheterotrophic, saccharolytic metabolism with a relatively narrow spectrum of carbohydrates supporting growth. From the tested polysaccharides, the most active growth was observed on alginate. Weaker growth (with highly aggregated cells)—on soluble starch (alpha-glucan) and inulin (β-fructan)—was observed. Mannose and fucose supported excellent growth, while growth on glucose, fructose, sucrose, melizitose, cellobiose, and maltose was less vigorous. The type strain (in contrast to AB-alg4) also grew with melibiose and raffinose, but the cells showed signs of osmotic stress (being swollen and lysing rapidly). During growth on all these substrates, cells were highly aggregated. The following polysaccharides tested negative as substrates: agarose, gellan, heparin sulfate, chondroitin sulfate, fucoidan, hyaluronan, pectin (citrus or apple), polygalacturonate, rhamnogalacturonan, starch, dextran, beech xylan, xyloglucan, arabinan, galactan, amorphous chitin, cellulose, and β-mannan. The monosugars, alcohols, and organic acids tested but not utilized included arabinose, galactose, rhamnose, raffinose, trehalose, lyxose, xylose, lactose, ribose, glucosamine, *N*-acetylglucosamine, mannitol, acetate, propionate, pyruvate, lactate, succinate, malate, and fumarate. Anaerobic fermentative growth occurred with mannose and alginate. Yeast extract and peptones from meat or casein neither supported growth as sole substrates nor showed stimulatory effect on growth with alginate.

Anaerobic respiration was tested with 4 mM nitrite as the electron acceptor and alginate or mannose as carbon and energy sources in comparison with the fermentative growth without nitrite. The results were positive only in the case of alginate: nitrite was completely consumed with the final growth yield doubled compared to fermentation. The product was nitrous oxide (N_2_O), as shown by gas chromatography. Consumption of 4 mM nitrite resulted in the formation of 1.5–1.7 mM nitrous oxide in replicate experiments, close to the expected stoichiometry (2:1). Dissimilatory ammonification was not observed.

**Fig 3:**
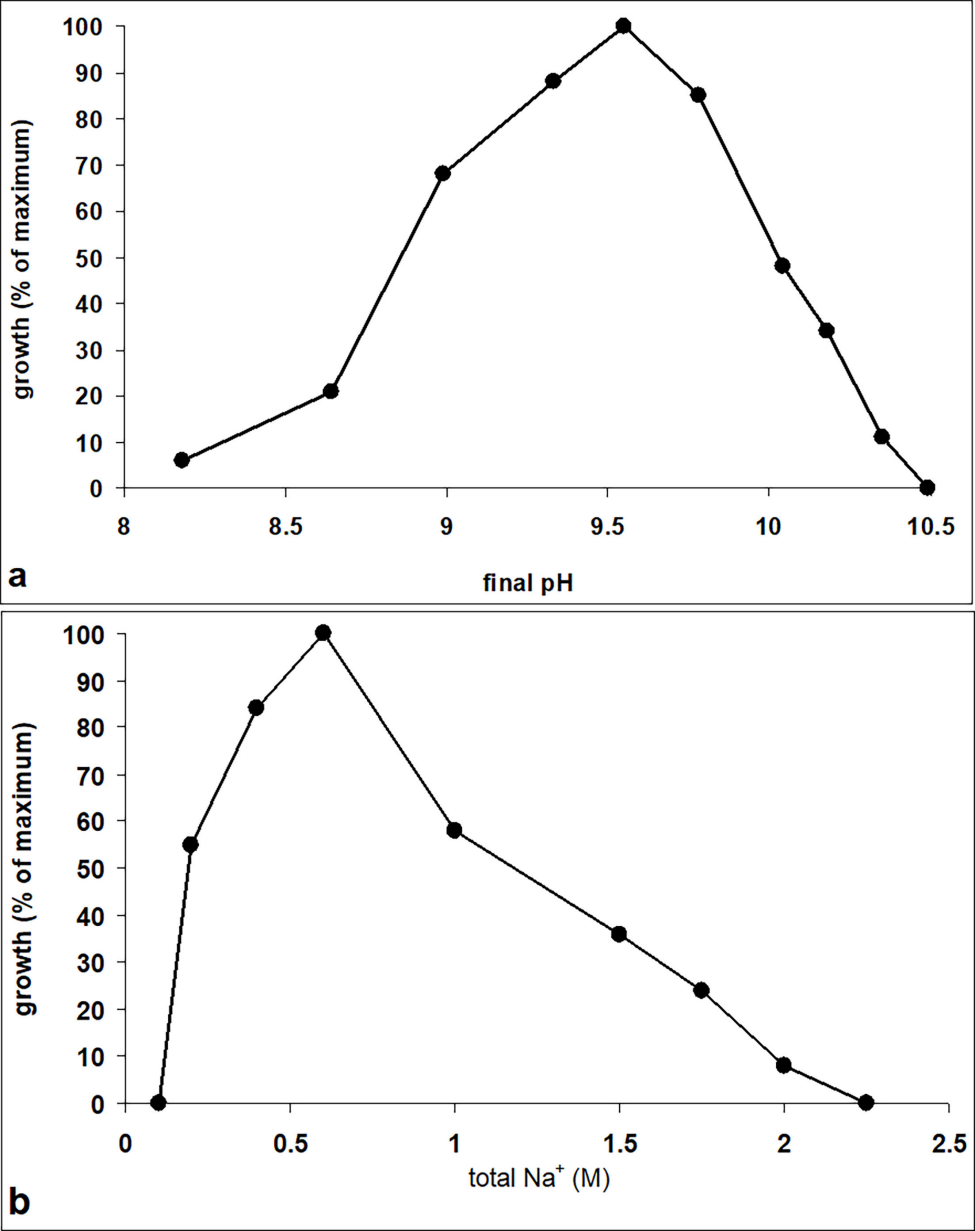
(**a**) pH (at 0.6 M Na^+^) and (**b**) salinity as sodium carbonates (at pH 9.5) for growth of strain AB-alg1^T^ with alginate at 30°C. The biomass was estimated by measuring OD_600_ (average values from a duplicate experiment).

Salinity (in the form of sodium carbonates) and pH (at 0.6 M total Na^+^) profiling (Fig. 3) characterized the new isolates as a moderately salt-tolerant obligate alkaliphile. The salinity range for aerobic growth on alginate was 0.3–1.8 M total Na^+^ (optimum at 0.4–0.6 M). The pH range that supported growth was from 8.2 to 10.3 (optimum at 9.5). The optimum growth temperature at pH 9.5 and 0.6 M total Na^+^ was 30°C with no growth above 40°C. A phenotypic comparison of AB-alg1^T^ and other *Opitutaceae* genera is presented in Table 1. What set the new isolates apart was their obligate haloalkaliphilic nature, which was unique within both the *Opitutales* order and the *Verrucomicrobiota* phylum as a whole. Note that previously reported growth of the *Opitutales* genera *Congregicoccus*, *Horticoccus,* and *Oleiharenicola* at pH 11 remains unsupported by actual pH measurements, while use of an unbuffered medium compromised conclusions (66–68). Another feature of the new isolates, distinguishing them from the majority of cultured members of *Verrucomicrobiota,* was their ability to use alginate as a growth substrate. Only hydrolysis of alginate, but not growth, has been reported previously in *Oceanipultrichrido coccoides* (69).

### Functional genome analysis of strain AB-alg1^T^

#### Alginate utilization potential

For the well characterized alginate-utilizing bacteria among marine *Bacteroidota* and *Gammaproteobacteria*, initial depolymerization of alginate is achieved by the concert action of various extracellular polysaccharide lyases (PL) of endo- and exo-types currently classified in PL families 6, 7, 12, 14, 15, 17, 18, 31, 34, 38, 39, and 44 of the CAZy database. The resulting alginate oligomers and dimers are further converted into unsaturated monouronate by glycoside hydrolases from the GH88 family located either in the periplasm or cytoplasm (14–18).

dbCAN and HMMER searches supplemented with Uniprot Blast predicted potential alginate lyases family proteins encoded in the genome of AB-alg1^T^, including PL6, 7, 12, 14, 15, 38, and 39, most with a Sec/SPI signal peptide, indicating their extracytoplasmic location (Table 2). Furthermore, a single GH88 family of unsaturated uronyl oligohydrolase (apparently cytoplasmic) was identified, as well as a potential Na^+^:oligoalginate symporter “ToaA” from the SSF superfamily (15). The alginate lyase encoding genes in the AB-alg1^T^ genome were not assembled into a canonical polysaccharide utilization locus (PUL), as is often the case with the alginate-utilizing bacteria, but, still, several of them were paired as a (endo-) + (exo-) types.

**TABLE 2:** Key functional proteins encoded in the genome of strain AB-alg1^T^ potentially involved in alginate metabolism^*a*^

Locus tag	CAZy PL family	Protein	Putative function	Signal peptide
Alginate depolymerization				
**000055**	PL39+CBM32/35/96	Alginate lyase	Endo-alginate lyase	Sec/SPI-SPII
**000060**	PL15		Exo-acting alginate lyase	Sec/SPI-SPII
000395	PL15		Exo-acting alginate lyase	Sec/SPII
**000404**	PL6_1		Versatile alginate lyase	Sec/SPI
**000406**	PL6		Versatile alginate lyase	Sec/SPI
**000879**	PL38		Endo-alginate lyase	(-) Globular
**001628**	PL17+CBM16/35/70		Versatile alginate lyase	Sec/SPI
**001646**	PL17+CBM6		Versatile alginate lyase	Sec/SPII
002984	PL7		Versatile alginate lyase	Sec/SPI-SPII
**003205**	PL38		Endo-alginate lyase	Sec/SPI-SPII
003733	PL17		Versatile alginate lyase	Sec/SPII
003800	PL17+CBM16		Versatile alginate lyase	Sec/SPI
**002469**	GH88	β-1,4 uronidase	Exo-β-1,4-alginate oligomer hydrolase	(-) cytoplasmic
**004163**				
Other polysaccharide lyases related to alginate lyases				
**000048**	PL29	Chondroitinase	Chondroitin sulfate ABC/hyaluronan	Sec/SPI
**000052**	PL29	Chondroitinase	Chondroitin sulfate ABC/hyaluronan	Sec/SPI
**001723**	PL21	Heparinase II	Heparin sulfate	Sec/SPI
**001725**	PL21	Heparinase II	Heparin sulfate	Sec/SPI
002230	PL8_3	Hyaluronidase	Hyaluronan	Sec/SPI
002245	PL35	Chondroitinase	Chondroitin sulfate (in bacteria)	Sec/SPI
002257				(-) Globular
002268				(-) cytoplasmic
Transport of the alginate hydrolysis products				
**001433**–**001438**	TonB/2 × TolQ/ExbB/ TolR/ExbD/TonB plug	**OM channel**/ **OM transporter**/ Energy transducer	TonB-dependent transport system: outer membrane transport of oligomers	Sec/SPI
				Sec/SPIII
				Membrane
**004062**–**004064**	TolQ/ExbB + TolR/ExbD/TonB plug	**OM transporter**/ Energy transducer		
**004214**– **004215**	TolQ/ExbB + TolR/ExbD	**OM channel** **OM transporter**		
**000051**	SglT/ToaA	Na^+^ symporter SSF	Putative uronate transporter	CPM
Cytoplasmic uronate metabolism				
**004145**	DehR	NADPH-dependent oxidoreductase	4-deoxy-L-erythro-5-hexoseulose uronate>2-keto-3-deoxy-D-gluconate	
**000255**	KduD/GndA	2-keto-3-deoxygluconate oxidoreductase	DehR homolog	
**001079**				
003637				
**000038**	KdgK	Sugar phosphokinase	2-keto-3-deoxy-D-gluconate> 2-keto-3-deoxy-6-phosphogluconate	
003081				
**003506**				
**000037**	KdgA/KDGP aldolase	Sugar aldolase	2-keto-3-deoxy-6-phosphogluconate> pyruvate + glycero-3-phosphate	
**002471**				
003082				
**002140**	KduI/UxaC	Uronate isomerase	Glucuronate><fructuronate	
**002468**	KdgF KdgR/GntR	Cupin Cupin+HTH-3	Putative pectin-utilization protein transcriptional regulator of the Kdg/Kdu pathway	
**002470**				
000254	UxuA	Mannonate dehydratase	Mannonate>2-keto-3-deoxy-D-gluconate	
003636				
000517				
000518	UxuB	Mannonate dehydrogenase	Mannonate>fructuronate	
000519	UxaC/KduI	Uronate isomerase	Glucuronate><fructuronate	

^*a*^ CBM, carbohydrate binding module. In bold: proteins expressed during growth on alginate, mannose, or sucrose. Gray shading indicates proteins upregulated during growth on alginate relative to growth with mannose or sucrose. See also Fig. 4 and Table S3.

**Fig 4:**
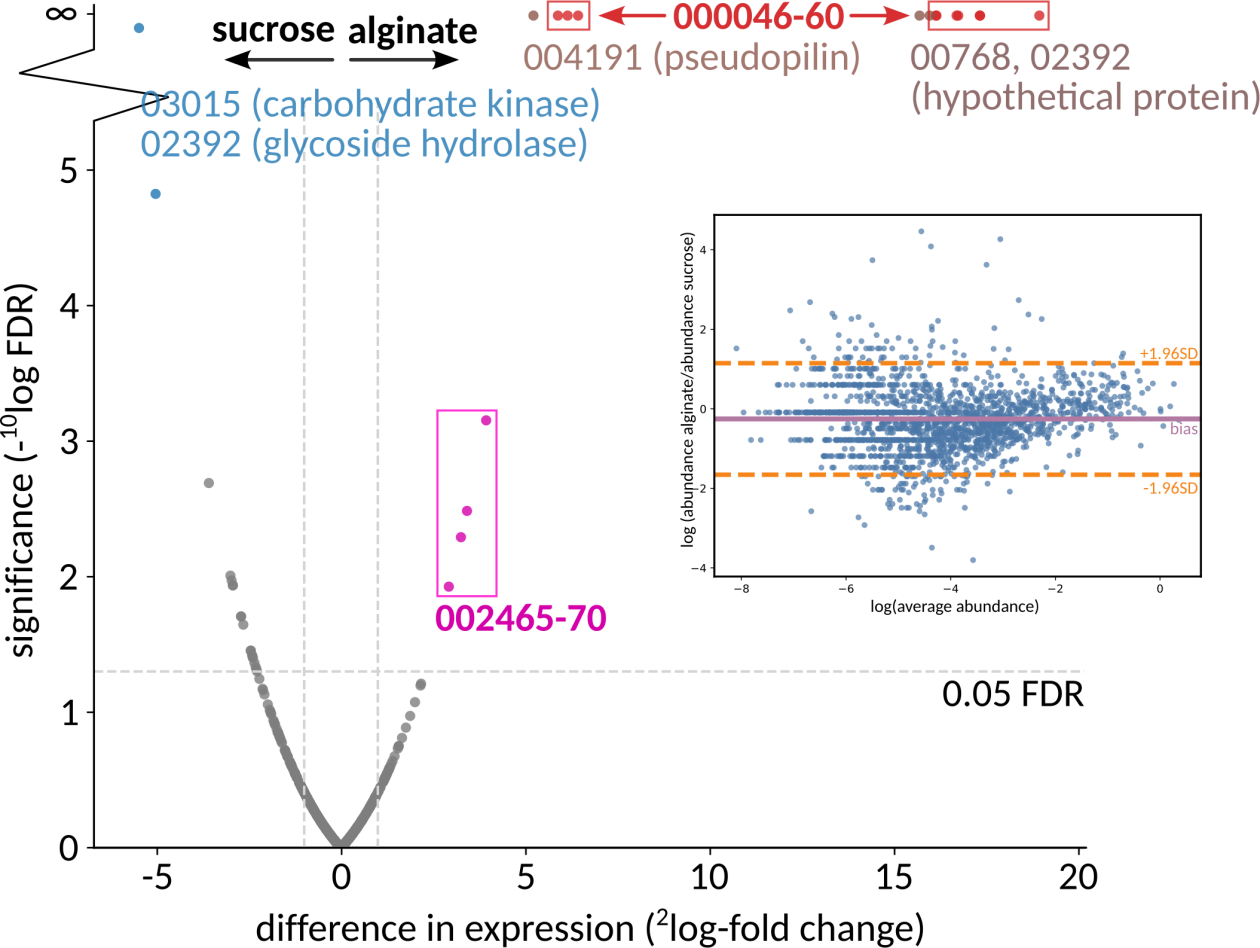
Volcano plot showing differences in expression between sucrose (left) and alginate (right). Dotted lines show thresholds for fold-change (*x* axis) and significance (*y* axis). Proteins part of the identified alginate degradation gene clusters are shown in red and pink. Gene identifiers are indicated for differentially expressed proteins. Only proteins with a relative abundance of at least 0.05% in either treatment are shown (*n* = 685). Because we had to work with one replicate proteome per treatment, we estimated the standard deviation assuming that expression of most proteins was independent of the treatment. The (inset) Bland-Altman plot shows that the standard deviation was independent of the mean after log transformation of the data, so significance could be estimated this way.

The known pathway of alginate metabolism in *Gammaproteobacteria* consists of first importing alginate oligomers through the outer membrane via porins (TonB or SusCD systems) (70). Next, final hydrolysis of the oligomers into C6-uronate—4-deoxy-L-erythro-5-hexoseulose uronic acid, DEHU—is performed by GH88 unsaturated uronyl hydrolase either in the periplasm or in the cytoplasm. Finally, reductive cleavage of DEHU into pyruvate and 3-phosphoglycerate is performed by the Kdu-Kdg system (15–17). Homologous proteins similar to all the enzymes involved in this pathway were identified in the AB-alg1^T^ genome, including a TonB-dependent outer membrane porin, GH88, DEHU reductase (DehR), 2-dehydro-3-deoxy-phosphouronate kinase (KdgK), and 2-keto-3-deoxy-6-phosphouronate aldolase (KdgA, part of the Entner-Doudoroff pathway). In some cases, multiple similar proteins were present in the genome. To test if the pathway was expressed in the presence of alginate, we grew strain AB-alg1^T^ with alginate, sucrose, and mannose in parallel incubations, extracted and digested protein, and analyzed expression using Orbitrap mass spectrometry. Many of the identified proteins were highly expressed and upregulated in the presence of alginate compared to growth on mannose and sucrose (Fig. 4 and Table 2).

The genome featured two clusters of genes potentially involved in alginate degradation that appeared to be upregulated as single operons in response to alginate: pgaptmp_000046-60 and pgaptmp_02465-70 (Fig. 4; Table S3). The former appeared to be dedicated to depolymerization and import, containing endo- and exo-PL families 15 and 39 alginate lyases, two PL29 chondroitinases, and a putative uronate transporter, among other genes (Table 2). The latter appeared to be dedicated to the further degradation of the monomers in the cytoplasm and contained β-1,4 uronidase, KdgA/KDGP aldolase/putative pectin-utilization protein, and a transcriptional regulator of the Kdg/Kdu pathway. Out of the 17 genes that were significantly over-expressed on alginate compared to sucrose, 14 were part of those two gene clusters (Fig. 4; Table S3). The three other over-expressed genes encoded a pseudopilin and two hypothetical proteins.

**Fig 5:**
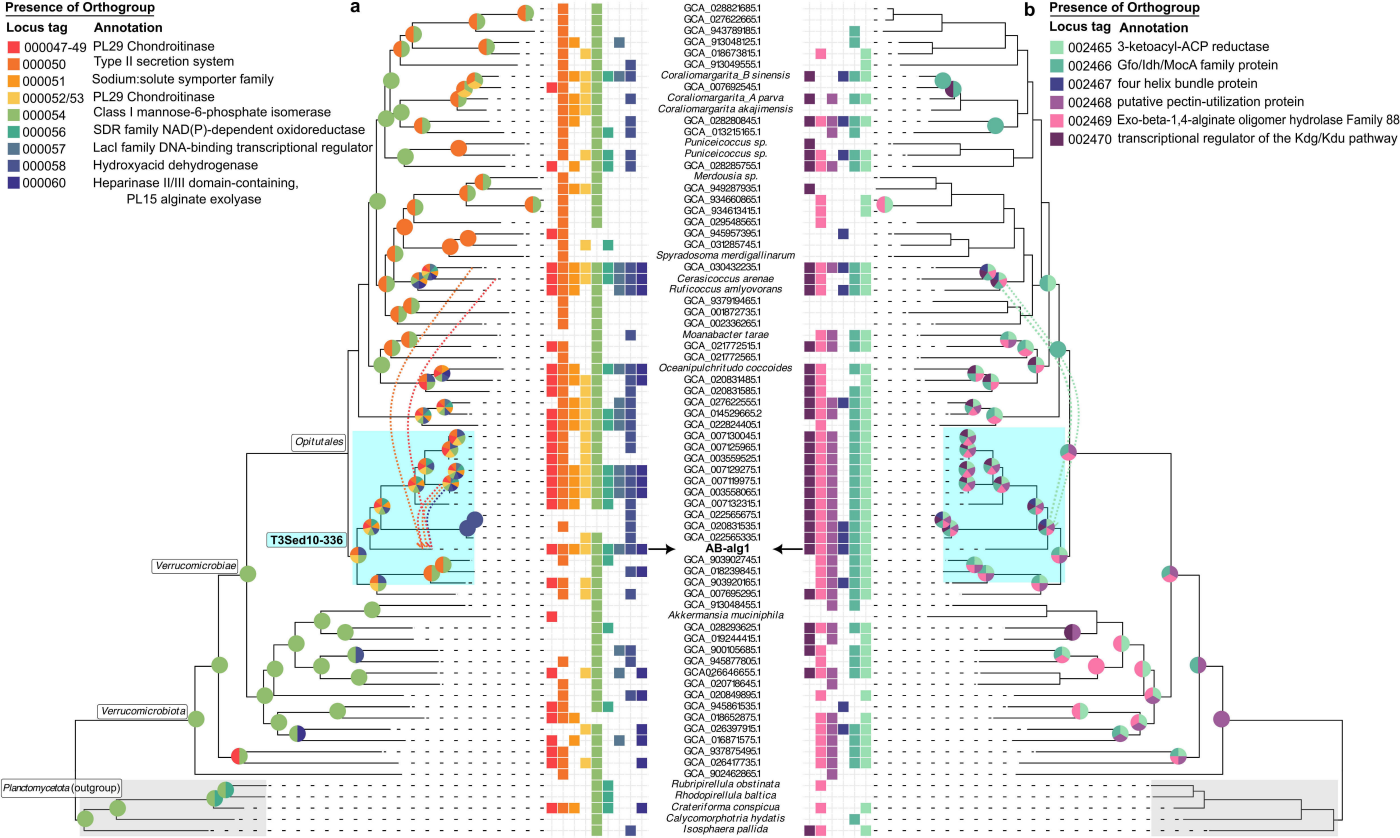
Reconciled species tree with presence of orthogroups associated with operons pgaptmp_000046-60, responsible for alginate depolymerization (**a**) and pgaptmp_02465-70, responsible for monomer degradation (**b**). Heatmaps show which orthogroups are present in the leaf genomes, while pie charts show which orthogroups are present at the ancestral nodes. Orthogroups associated with locus tags are given in Table S5. T3Sed10-336 is highlighted in cyan. Dashed arrows show horizontal gene transfer of [pgaptmp_000046-50, 60] from other T3Sed10-336 bacteria and [pgaptmp_000047–50, 2465] from *Cerasicoccaceae*. Conversely, ancestors of AB-alg1^T^ may have transferred [pgaptmp_002466] to *Cerasicoccaceae*. Note that *Cerasicoccaceae* have no current species adapted to alkaline environments.

To explore the evolutionary history of these gene clusters, phylogenetic trees of genes from both upregulated regions were reconciled with the species tree using AleRax (40). Ancestral reconstruction (Fig. 5) showed that homologs of the alginate degradation genes from the gene cluster [pgaptmp_000047–000060] occur in all T3Sed10-336 MAGs from Kulunda soda lakes, but not in those from Canadian soda lakes (GCA_022565335, GCA_022565675, and GCA_020831535). It is possible that AB-alg1’s Canadian relatives feed on a different, related polysaccharide. Among other *Opitutales*, [pgaptmp_000047–000060] was only (nearly) complete in *Oceanipulchritudo coccoides,* a known alginate consumer (69), and *Cerasicoccaceae*. A broader search for polysaccharide lyase gene families among *Opitutaceae, Oceanipulchritudinaceae,* and T3Sed10-336 suggested that the use of alginate and related acidic polysaccharides might be widespread among all three families (Table S4). So far, growth with alginate was not tested in any of the isolated species and only a single organism tested positive for alginolytic activity (*Oceanipultrichido*). Future research might investigate the use of alginate more systematically for members of the *Opitutales*. The most well represented alginate lyase type among the analyzed genomes was PL38, which is also widespread among marine *Verrucomicrobiota* (71).

AB-alg1^T^ had some unique features contributing to its alginolytic potential. First of all, the upregulated PL39 endoalginate lyase (pgaptmp_000055) did not have any close homologs among any of the genomes presented in Fig. 5 (although distantly related, <50% identity, proteins were present occasionally). Second, AB-alg1^T^ obtained additional copies of pgaptmp_000047–000050 and pgaptmp_000060 from other T3Sed10-336 bacteria as well as pgaptmp_000047–000050 from *Cerasicoccaceae*. For the pulG-like type II secretion pseudopilin, pgaptmp_000050, no less than 67 homologous proteins were present in the AB-alg1^T^ genome, obtained in six gene-transfer events (Fig. S1 and S2), with remaining copies attributed to gene duplication. The type II secretion system (pgaptmp_004181-004193) could be involved in the export of the alginases pgaptmp_000055 and 000,060, as was previously shown for cellulases in *Cytophaga hutchisonii* (72).

#### Other predicted key functional properties

The AB-alg1^T^ genome, as well as the genomes of related *Opitutales* (Table S4), encode multiple putative alginate lyases and also other PL families with potential hydrolytic activities against acidic glycosaminoglucan polysaccharides. Hyaluronate, chondroitin, and heparin (PL 8, 29, 33, 35, 37) were most common. Furthermore, the AB-alg1^T^ genome encodes 67 copies of desulfatases or sulfohydrolases, mostly clustered in three large loci. Although such enzymes are essential for depolymerization of the sulfated glucosaminoglucans chondroitin and heparin, our growth tests of both AB-alg isolates gave negative results with these polysaccharides. An attempt to use these three polysaccharides as selective enrichment substrates resulted in isolates from completely different phylogenetic lineages. The ability of AB-alg strains to use the β-fructan inulin is supported by the presence of two GH 32 inulinase genes (one excreted, one cytoplasmic) in the AB-alg1ᵀ genome, while their growth on starch can be explained by multiple secreted GH13 amylases from subfamilies 10, 13, 14, 20, and 31.

As mentioned above, AB-alg1^T^ was able to grow by anaerobic respiration of nitrite (but not nitrate) to N_2_O with alginate as substrate. The genome encoded two types of dissimilatory nitrite reductase: a copper-containing denitrifying NirK (clade 5 in a bicistronic operon with its electron donor monoheme cytochrome *c*) and an ammonifying NrfAH. As we did not observe NH_3_ formation during anaerobic growth with nitrite, either with alginate or mannose as electron donors, the ammonifying pathway was apparently inactive, at least under the conditions tested. The denitrifying pathway from nitrite to N_2_O includes two enzymatic steps—nitrite reduction to NO by nitrite reductase (either Cu-containing NirK or cytochrome cd1 NirS) and NO reduction to N_2_O (either by quinol-dependent *e*NorB or cytochrome *c*-dependent cNorBC). The AB-alg1^T^ genome encodes a NirK plus its electron-accepting periplasmic cytochrome *c* and a homolog of the *e*Nor clade (52). This truncated denitrification pathway branches off a canonical aerobic respiratory chain consisting of Respiratory Complexes I–V. There were four heme-copper-family oxygen reductases, including high and low affinity types (Table 3), indicating that AB-alg1^T^ can cope with a wide range of oxygen concentrations.

**TABLE 3:** Proteins involved in haloalkaliphilic adaptation, energy metabolism, and structural functions encoded in the genome of strain AB-alg1^T^^*a*^

Locus tag	Protein	Putative function
Osmoprotection		
**000474**	Fused glycine/sarcosine/dimethylglycine *N*-methyltransferase	Biosynthesis of glycine betaine from glycine
003536	BetT	High affinity glycine betaine exporter
000665, 000704, 001269, 001482, **001618**, **003007**	Mechanosensitive small channel McsS	Osmoregulation
pH-ion homeostasis		
003463–003473	MrpABCD1D2**D3E**FG	Multisubunite sodium:proton antiporter
**000996**	CPA2	Na^+^ : H^+^ antiporter
**003406**	NhaC	Na^+^ : H^+^ antiporter
**004400;** 004402	TrkA**H**	Potassium uptake (K^+^ : H^+^ symporter)
000109	Kch	Volt-gate potassium channel
Primary sodium pumps		
**000786**; 004205	HppA	Sodium-translocating pyrophosphatase
002849–002851	**OadAB**G	Sodium translocating oxaloacetate decarboxylase
Aerobic respiratory chain		
000866–870/001048–53/001276–77	NuoN**M**LKJ/**BC**D**EF**G/H**I**	Complex I
003802–003813	NuoNM1M2LKJHIDC**B**A	
003232–003233	PetAB/C	Menaquinone-cytochrome *c* reductase Complex III
003234–003235	Cox I+III/II	Cytochrome *c* oxidase *caa_3_* (1) Complex IV
**003962–003968**	ActABCDEF	Alternative complex III
**003969–003971**	Cox I–IV	Cytochrome *c* oxidase *caa_3_* (2) Complex IV
002460–002464	CtaB**СD**EG	Cytochrome *c* oxidase *caa_3_* (3) Complex IV
002561–002568	CcoIGQ**PN**S	Cytochrome *c* oxidase *cbb_3_* Complex IV
**001090–001097**	AtpεβγαΔ/BCA	H^+^-translocating F1F0 ATP synthase
Anaerobic respiration		
002513–002514	Cytochrome *c*/ NirK	[Cu] dissimilatory nitrite reductase (clade 5)
001242–001243	eNorAB	Dissimilatory NO-reductase
003174–003175	NrfAH	Dissimilatory ammonifying nitrite reductase
Structural elements		
**000266**	L-fuculose phosphate aldolase FucA/RhaD	BMC (Biological MicroCompartments/encapsulins) locus
000267, **000268**, **000270**	EutN/CcmL microcompartment shell vertex protein	
**000271, 000273, 000274**	EutM/CcmK/PduA microcompartment shell protein	
**000269**	Propionaldehyde dehydrogenase EutE/PduP	
**000272**	Acetate/propionate kinase EutQ/PduW	

^*a*^ In bold: proteins expressed in cells growing aerobically with alginate. The complete data are provided in Table S3.

#### Haloalkalphilic adaptation, energy generation, and structural features

A search for genes encoding biosynthesis of osmolytes identified a single candidate gene encoding a fused version of the two SAM-dependent *N*-methyltransferases catalyzing step-wise N-methylation of glycine to glycine betaine (73). In addition, external glycine betaine could be imported by a high-affinity BCC transporter BetT. AB-alg1^T^ may also use mechanical osmoregulation, based on the presence of six copies of genes coding for mechanosensitive small channel proteins McsS (Table 3). With respect to pH and ion homeostasis, the AB-alg1^T^ genome features the multisubunit Na^+^: H^+^ antiporter Mnh/MrpEFGBCD1D2D3, two single-subunit Na^+^: H^+^ antiporters CPA2 and NhaC, and a K^+^: H^+^ symporter TrkAH. Furthermore, genes for two primary sodium pumps, including two copies of a sodium-translocating pyrophosphatase HppA and a sodium-translocating oxaloacetate decarboxylase OadABG, were identified. The latter is often present in anaerobic alkaliphilic bacteria (74).

A large genomic locus (10 genes) containing marker for biological microcompartments (BMCs) (75) or encapsulins was present in the genome of AB-alg1^T^ (Table 3) and was nearly identical in composition to one found in the hyaluronan-utilizing planctomycete *Natronomicrosphaera* (13). Such protein shells are formed to enclose toxic metabolic pathways in bacteria, in particular toxic aldehydes and alcohols during glycolytic degradation of rhamnose and fucose (76, 77). Thin sectioning did not show the typical BMC-like inclusions in the two isolated soda lake bacteria, at least when the cells were growing on their respective acidic polysaccharides.

### Conclusion

Strain AB-alg1^T^ and AB-alg4 are the first pure culture representatives of natronophilic *Verrucomicrobiota,* isolated from soda habitats, and, to the best of our knowledge, also a first documented case of *Verrucomicrobiota* capable of growth on alginate. Nonetheless, the results of comparative genomic analysis showed that several *Opitutales* members do have alginolytic potential and that genes upregulated for alginate degradation in AB-alg1^T^ might have been transferred from other lineages. Future research should explore the utilization of alginate as a growth substrate in culturable representatives of this phylum, especially those from marine habitats that provide for abundant alginate.

Based on distant phylogenomic and unique phenotypic properties, the natronophilic alginotrophic strains AB-alg1^T^ and AB-alg4 from soda lakes and soda solonchak soils, respectively, are proposed to be classified into a new genus and species *Verruconatronum alginivorum* gen, nov., sp. nov. within a new family *Verruconatronaceaea* fam. nov., which also incorporates several genus-level groups of MAGs from similar soda lake habitats, currently known as TS3-sed10 in GTDB. *Verruconatronaceae* forms a sister family to *Opitutaceae w*ithin the order *Opitutales.*

### Description of *Verruconatronaceae* fam. nov.

[Ver.ru.co.na.tro.na'ceae. N.L. neut. n. *Verruconatronum*, the type genus of the family; -*aceae* ending to denote a family; N.L. fem. pl. n. *Verruconatronaceae,* the family of the genus *Verruconatronum*]

The family *Verruconatronaceae* includes facultatively anaerobic, moderately salt-tolerant, and obligately alkaliphilic organoheterotrophic bacteria living in aquatic and terrestrial haloalkaline habitats. The members are saccharolytic, utilizing polysaccharides and simple sugars for aerobic or fermentative growth or anaerobic growth by denitrification. A particularly characteristic substrate is alginate from brown algae. The family includes a type genus *Verruconatronum* consisting of cultivated species and three genus-level groups comprised of MAGs from soda lakes in southwestern Siberia and Canada. The family is a member of the order *Opitutales*, class *Opitutia*, phylum *Verrucomicrobiota*.

### Description of *Verruconatronum* gen. nov.

[Ver.ru.co.na.tro'num. L. fem. n. *verruca*, a wart; N.L. neut. n. *natron*, soda (arbitrarily derived from the Arabic n. *natrun* or *natron*); N.L. neut. n. *Verruconatronum*, soda-loving *Verrucomicrobiota* genus]

The genus includes moderately salt-tolerant, alkaliphilic, facultatively anaerobic, heterotrophic bacteria specialized on utilization of alginate as growth substrate. They are small motile cocci without pigmentation. Currently, it includes a single species based on two pure cultures isolated from soda lakes and soda solonchak soil. The genus is classified within its own family *Verruconatronaceae*, order *Opitutales*. The type species is *Verruconatronum alginivorum*.

### Description of *Verruconatronum alginivorum* sp. nov.

[al.gi.ni.vo'rum. N.L. neut. n. *acidum alginicum*, alginic acid; N.L. neut. adj. suff. -*vorum*, devouring; from L. v. *voro*, to devour; N.L. neut. adj. *alginivorum*, alginic acid-devouring]

Cells are cocci, 0.7–1.0 μm, motile by 1–2 flagella with the Gram-negative type of the cell wall ultrastructure. The colonies are hard, white, up to 2 mm, forming inside of soft agar with concentration 0.6%–0.8%. The polar membrane phospholipids are dominated by phosphatidylcholine and diphosphatidylglycerols (cardiolipins). Minor components include phosphatidylethanolamines, lyso-phosphatidylcholines, phosphatidylglycerols, and its acyl derivative. The dominant polar lipid fatty acids (in order of abundance) are *anteiso-*15:0, 18:1ω9, 18:0, and 16:0. The only respiratory lipoquinone is MK-7:7. It has the genetic potential to synthesize the osmolyte glycine betaine from glycine and to produce Bacterial MicroCompartments (BMC). It is a facultatively anaerobic saccharolytic bacterium growing best with alginate and mannose. It can also utilize starch, β-fructan inulin, and simple sugars, including glucose, fructose, fucose, sucrose, maltose, cellobiose, and melezitose. Anaerobic fermentative growth was possible with alginate and mannose and anaerobic respiration—with nitrite as the *e*-acceptor (N_2_O as the final product). Ammonium, urea, and nitrate serve as the nitrogen source. It is moderately salt-tolerant with the total Na^+^ (as sodium carbonates) range from 0.2 to 1.8 M (optimum at 0.4–0.6 M). Obligate alkaliphilic with a pH range for growth from 8.2 to 10.3 (optimum at 9.5). Mesophilic, with the upper temperature limit for growth (at optimal pH and salinity) at 40°C. The G + C content of the genomic DNA is 59.4%. The type strain, AB-alg1^T^ (JCM 35393^T^=UQM 41574^T^), was isolated from a mix sample of surface sediments and brines from soda lakes in Kulunda Steppe (Altai, Russia) and an additional strain AB-alg4 (identical to the type strain in its 16S rRNA gene sequence)—from soda solonchak soils in the same area. The genome size of the type strain is 6.01 Mb.

## Data Availability

The DNA sequencing data were deposited in the NCBI’s Short Read Archive (SRA) under accession numbers SAMN53477254 (Biosample) and SRS27444179 (read sequences). The GenBank 16S rRNA gene sequences of AB-alg1^T^ and AB-alg4 are PX129057 and PX129056, and the assembled genome sequence of the type strain was deposited under accession number GCA_056590205. The proteomic data were deposited in the proteomic archive PRIDE under accession number PXD071159.

## SUPPLEMENTAL MATERIAL

Supplemental figures aem.00475-26-s0001.pdf Fig. S1 and S2.

Supplemental tables aem.00475-26-s0002.xlsx Tables S1 to S6.

ASM does not own the copyrights to Supplemental Material that may be linked to, or accessed through, an article. The authors have granted ASM a non-exclusive, world-wide license to publish the Supplemental Material files. Please contact the corresponding author directly for reuse.
