# Supplementary material for: Physiology, functional genomics, and proteomics of Verruconatronum alginivorum gen. nov., sp. nov., the first isolated haloalkaliphile within Verrucomicrobiota, representing a new family, Verruconatronumaceae fam. nov
Source: Appl Environ Microbiol. 2026 May 11;92(6):e00475-26. doi: 10.1128/aem.00475-26 (PMC13274419; doi:10.1128/aem.00475-26)
Supplement: Supplemental figures — Fig. S1 and S2. [file aem.00475-26-s0001.pdf]

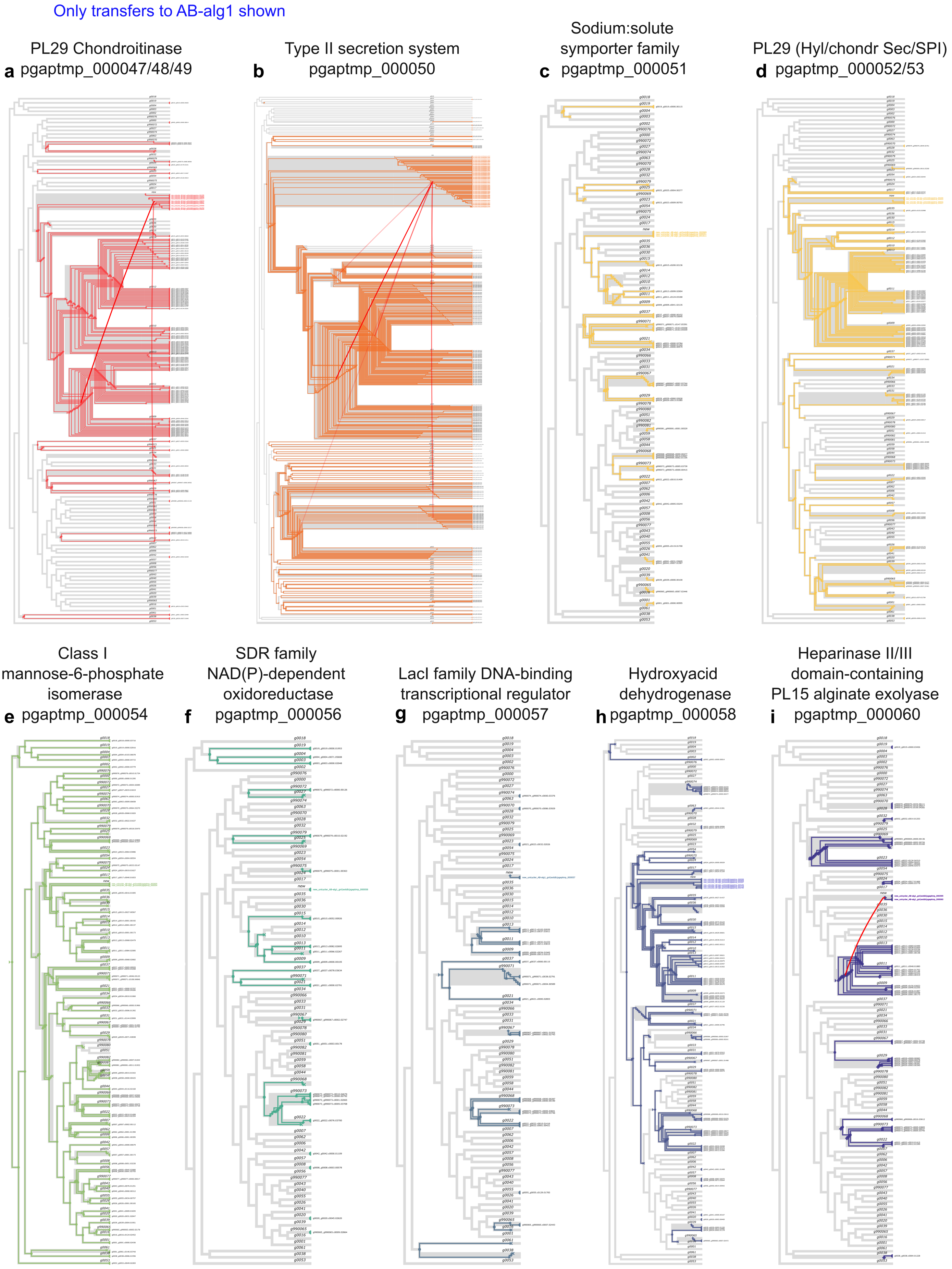

Figure S1. Ancestral reconstruction of pgaptmp_000046-60, showing transfer of several genes into the AB-alg1T lineage. For each gene, colored line show co-speciation of genes and species.

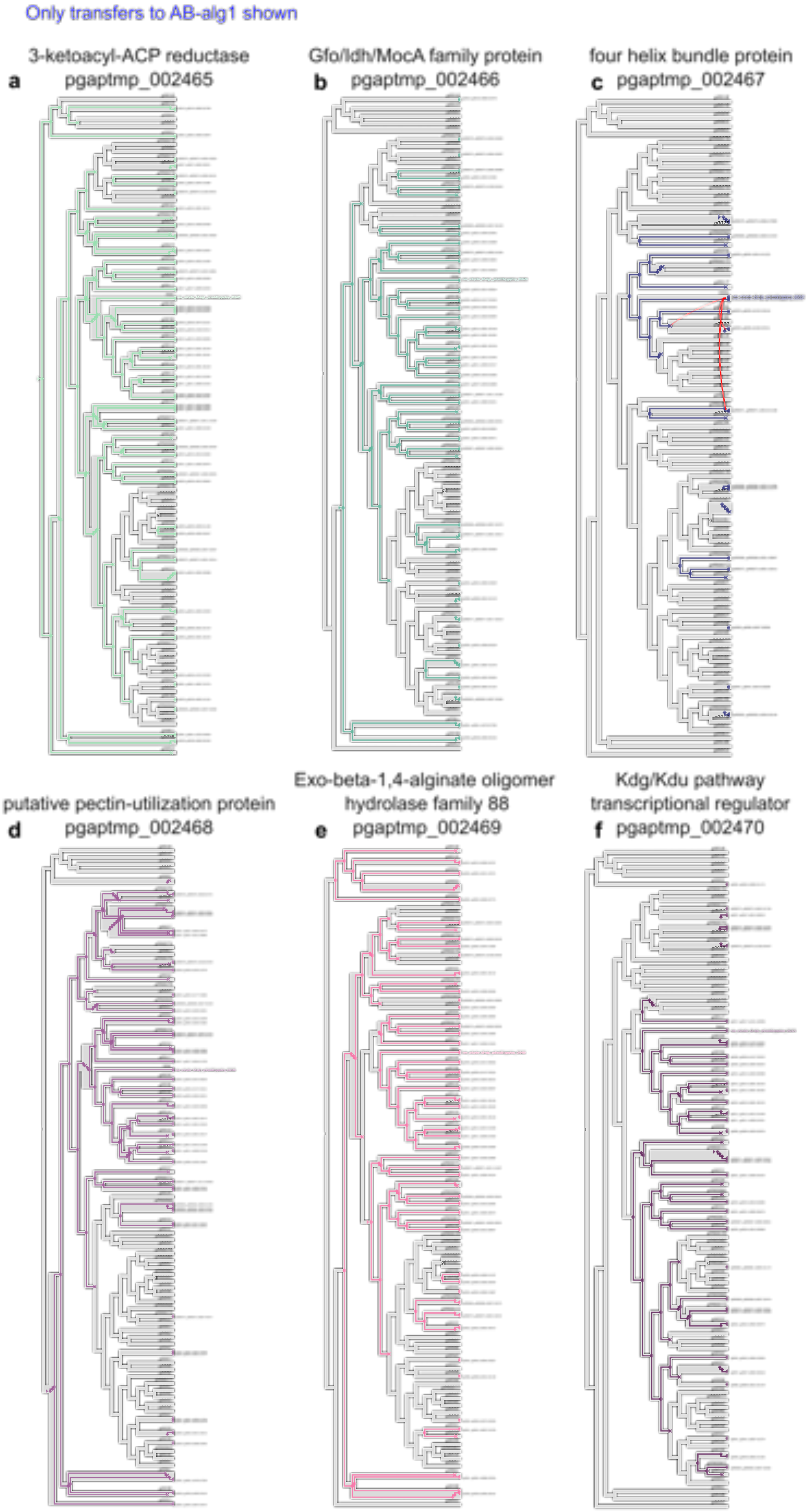

Figure S2. Ancestral reconstruction of pgaptmp_02465-70, showing transfer of pgaptmp_02467 into the AB-alg1T lineage. For each gene, colored line show co-speciation of genes and species.
